# Designing and developing a co-produced theoretical and evidence-based online support for family caregivers of people with dementia at the end of life

**DOI:** 10.1186/s12904-019-0455-0

**Published:** 2019-08-13

**Authors:** Nathan Davies, Jenny Hopwood, Nina Walker, Jamie Ross, Steve Iliffe, Kate Walters, Greta Rait

**Affiliations:** 1 Centre for Ageing Population Studies, Research Department of Primary Care and Population Health, University College London, Rowland Hill Street, London, NW3 2PF UK; 2 Centre for Dementia Palliative Care Research, Marie Curie Palliative Care Research Department, Division of Psychiatry, University College London, 149 Tottenham Court Road, London, W1T 7NF UK; 3 eHealth Unit, Research Department of Primary Care and Population Health, University College London, Rowland Hill Street, London, NW3 2PF UK; 4 Priment Clinical Trials Unit, Research Department of Primary Care and Population Health, University College London, Rowland Hill Street, London, NW3 2PF UK

**Keywords:** Dementia, Palliative care, Caregivers, Internet, Digital intervention development, Co-design

## Abstract

**Background:**

Caring for someone with dementia can be physically and emotionally difficult. Acting as a caregiver can make it difficult to access sources of support, particularly in the later stages of dementia. This paper reports the development and presents the targets (subject areas) and components of a prototype website to support family caregivers of a person with dementia towards the end of life.

**Methods:**

Adopting an iterative approach and co-production methods the development process consisted of four stages: **Stage1-Synthesis of data:** three sources of data (interviews, systematic review and theory) were synthesised using tabulation, to identify the targets of the prototype; **Stage2-Identifying intervention targets and components:** a research development group (health practitioners, a family caregiver and academic experts) met to discuss the development, using a modified nominal group process, refining the synthesis from stage 1; **Stage3-Developing the intervention prototype:** an outline of the prototype was developed based on stage 1 and 2; and **Stage4–User testing:** interviews with caregivers testing the prototype website.

**Results:**

Qualitative interviews with caregivers identified four targets for the intervention: 1) feeling prepared and equipped; 2) feeling connected and supported; 3) valuing themselves as a caregiver and as an individual; 4) maintaining control of the caring situation and being the coordinator of care. The systematic review provided evidence on how and what components could address these targets, including providing information, peer support, contact with professionals, and psychological support. Theory helped to narrow the focus within each of these targets. Active discussion with the research development group and end users provided an outline of the prototype website. The prototype website presented addresses these targets with written information, videos from other caregivers, and peer and professional support sections. The subject areas covered included expectations at the end of life, support with day-to-day caring, care planning, and communication.

**Conclusions:**

This paper provides a detailed account of the development process of a prototype website for caregiver support. The transparent methodology and key lessons learnt from developing the prototype should help those who are developing similar interventions, across complex, progressive conditions and not just limited to dementia.

## Background

Around two thirds of people with dementia live in the community [1] with the majority of their care provided by friends or family, referred to as caregivers for this paper. Without the help of such caregivers the formal care system would likely collapse [2]. Policy in the UK and internationally supports the provision of ‘informal’ care from caregivers, in order to support health and social care systems [3]. Caring for someone with dementia is considered one of the most stressful and difficult forms of caring [4]. The effects are evidenced in both physical and psychological ill health of caregivers, including higher levels of strain and depression, and in some instances premature mortality [5–8].

Caring towards the end of life can be particularly challenging as medical symptoms and complications increase and the person becomes less responsive and able to communicate [9, 10]. Caregivers have reported a perceived gap in support provided from services for the challenges they face towards the end of life, such as caring at home and navigating complex care systems [11–13]. The strain on health and social care services worldwide has resulted in a shift from formal care services providing care, to caregivers providing more care for longer at home [14, 15]. It is therefore imperative that resources are in place to support caregivers in this role.

Use of digital resources including websites to meet the needs of patients and family caregivers is a growing area of research and policy development, and provides an opportunity to close this ‘support gap’, particularly for caregivers finding it difficult to leave their home due to caring responsibilities [16, 17]. A systematic review of the literature of existing internet-based interventions (mainly websites) aimed at supporting family caregivers of people with dementia, identified that such interventions have the capacity to improve various aspects of caregiver well-being, including depression, anxiety and burden [14] and this has been shown in additional reviews of older adults more generally [18–21].

Use of the internet by older adults is growing, with an increase in over 20% in recent internet usage among the 65–74 years age group [22]. These figures are likely to rise as younger age groups already familiar with technology age. Qualitative data suggests caregivers have positive views of receiving support online via a website, and help support them with when caring for someone with dementia approaching the end of life [23, 24].

This paper reports the development and presents the core targets (subject areas) and components of a prototype website to support family caregivers of a person with dementia towards the end of life. The main study consisted of three phases to develop the prototype website: phase 1) evidence synthesis and mapping exercise of existing resources; phase 2) qualitative study with family caregivers of people with dementia towards the end of life; phase 3) synthesis of data from phase one and two to develop a prototype website and user testing. Results from phases one and two have been published previously [16, 23], this paper reports on phase 3 which was split into four separate stages.

We adopted a broad view to define end of life care similar to that of the European Association of Palliative Care which define it is an extended period in which professionals and families become aware of the life limiting nature of the illness and acknowledge the person is dying [25]. As a study developing an intervention for family caregivers, we aligned our view of end of life care with theirs based on previous work and in earlier phases of data collection in the current study. This is a period in which there is increased dependency and physical decline of the individual and not time specified [9, 23].

## Aim

This paper reports the development and presents the core targets and components of a prototype website to support family caregivers of a person with dementia towards the end of life.

The specific objectives are to:

1. Describe the development process and synthesis of different data
2. Present the core targets and components of the prototype website
3. Further develop the prototype with end users through user testing

## Methods

### Design

**Fig. 1:**
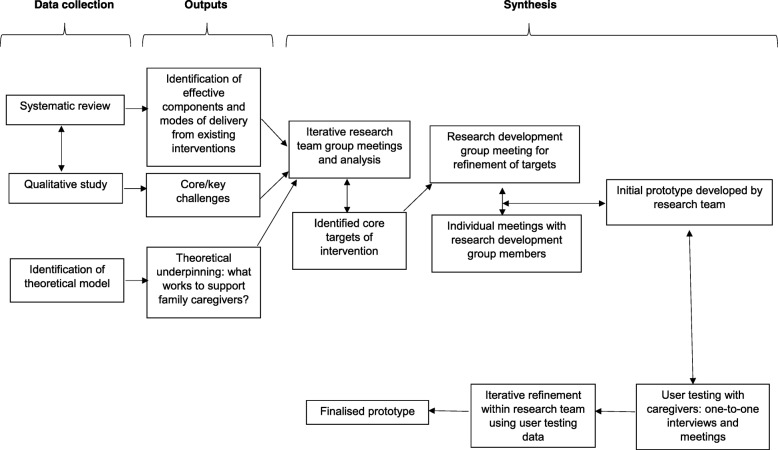
Overview of prototype development and synthesis

This study adopted an iterative co-production method for intervention development [26–28], following the MRC framework for developing a complex intervention [29]. An overview of the procedure for the whole study and synthesis process for developing the prototype website is shown in Fig. 1.

### Caregiver support theory

The development of the prototype website was supported by a published ‘realist’ theoretical framework which was developed through investigating ‘what works to support family caregivers of people with dementia’ [30]. The underpinning mechanism of the theory is that resilience and hence resilience building are central to what works to support family caregivers of people with dementia. The cohesive mechanism consists of five domains which together interact to increase resilience. These include: extending social assets; strengthening key psychological resources available to caregivers; maintaining caregiver’s physical health status; safeguarding caregiver’s quality of life; and ensuring timely availability of key external resources. The theory provides potential mechanisms within each of the five domains and explains how they may operate to increase resilience and support for family caregivers. Table 1 demonstrates how these domains relate to the themes from the interview data and the systematic review’s findings. The theory helped to narrow the focus within each of these themes, which became the targets of the website, and hence provided an outline of the prototype website.

**Table 1:** Mapping data, theory and systematic review to prototype intervention

Output		Input		
Target of the intervention	Intervention components (topics and sections)	Qualitative data	Key theoretical themes	Systematic review
Feeling prepared and equipped	What can I expect towards the end of life? (Fig. 3)	Caregivers reported a lack of information particularly about end of life care. Caregivers discussed a desire and need to feel prepared. There was a need to feel more confident and knowledgeable about how to manage the medical decline.	• Ensure timely availability of key external resources	Individualised information was considered more beneficial by caregivers and thought to be one of the most useful components of interventions. The section was developed to be engaging through the use of a video from a caregiver which families could relate to and creating a sense of peer support which was highlighted as important in the systematic review.
	Day to day caring (Fig. 4)			
	Planning (Fig. 5)			
Feeling connected and supported	Chat to a caregiver (Fig. 6)	Participants described caring as a lonely experience in which they often felt socially isolated, and wanted to feel both connected and supported by other caregivers and professionals.	• Extending social assets	Support was able to be provided online though peer interaction and contact with professionals. Opportunities to engage with other caregivers in the same situation as them and in a group situation appeared more beneficial. Interaction with professionals was a positive experience for caregivers, however views were mixed on receiving this online.
	Talk to a professional (Fig. 7)			
	Communicating with the person with dementia (Fig. 8)			
Valuing themselves as a caregiver and an individual	Chat to a caregiver (Fig. 6)	In the interviews participants appeared to describe an internal conflict about caring for themselves and maintaining their own life, whilst caring. They needed mechanisms to cope with their emotions including; anger, grief and guilt.	• Strengthen key psychological resources available to caregiver; • Maintaining caregiver’s physical health status; • Safeguard caregiver’s quality of life	Several previous interventions included measures around the health and wellbeing of caregivers. Many interventions used psychological support, which demonstrated the most positive effect. Many used self-guided psychological support, most often consisting of educational modules. Randomised controlled trials (RCTs) found significantly greater improvements in stress, self-efficacy, intention to get support, strain, depression, and anxiety.
	Looking after yourself (Fig. 9)			
	Preparing for death (Fig. 10)			
Maintaining control of the caring situation being the co-ordinator care	Financial information (Fig. 11)	Caregivers felt they needed to take control and manage the care. Interspersed among these discussions was a lack of support from other family members and a lack of understanding from existing friends.	• Ensuring timely availability of key resources • Extending social networks	Several interventions identified provided local and tailored support or signposting of services, which was well received by caregivers. The internet resources may be a method of preparation for discussions, but decisions should be made face to face.
	Local support (Fig. 12)			
	Family relationships and social networks (Fig. 13)			

### Research development group

We created a research development group consisting of three academic experts in dementia, end of life, carers and digital health; health care practitioners (4 general practitioners, 1 academic nurse, 1 Admiral nurse); two members of a dementia charity organisation, and one caregiver. A second caregiver had to withdraw for personal reasons. The group had several roles starting with research design in phase 1 and 2 where the group helped to set the focus of the evidence synthesis and advised on data collection methods and questions for the interviews with caregivers. Finally, the group acted as a think tank to help interpret data from phase 1 and phase 2, acting as a first step in the co-production of the prototype website.

### User testing recruitment

Family caregivers (current and former) of people with dementia were recruited for individual interviews (*n* = 11) for user testing of the prototype. Participants were recruited through third sector organisations and Join Dementia Research (JDR). JDR is an online network of volunteers who are willing to take part in dementia research studies. Recruitment was supplemented with participants identified from interested general practices within London and Essex, the practices being identified through Clinical Research Networks. Older caregivers (65 years and above) were purposively sampled with a range of internet usage (no or minimal use of the internet, moderate use of the internet (e.g. weekly basis) and high use of the internet (e.g. daily basis), however it was difficult to recruit those with low usage. To ensure maximum diversity within the sample; gender, ethnicity/language and education were monitored. Experience of caring for someone with dementia at home towards the end of life was a requirement for participation.

Broad inclusion criteria were adopted to ensure a maximum response rate in a population which is often hard to recruit. Inclusion criteria consisted of caregivers currently caring or experience as a former carer (within the last three tears) for someone with dementia. Due to the difficulties of providing an accurate prognosis, defining the end of life time period for someone with dementia can be difficult [31], and so we did not impose a definition of end of life on caregivers. Participants were informed this was a study of end of life care and asked if they considered themselves eligible, having recently or previously cared for someone towards the end of life, as per previous studies [9]. Caregivers were excluded if they had experienced bereavement (death of person with dementia) within the last 3 months as they may find discussions around end of life distressing and may also be experiencing complicated grief. Those who had any cognitive impairment or were unable to provide informed written consent were also excluded.

### Procedure

An overview of the synthesis and development procedure for the prototype is shown in Fig. 1. Findings from the systematic review in phase 1 and qualitative data from phase 2 were used to inform the development of the prototype website.

The phase 3 development was split into four stages:

#### Stage 1 – synthesis of data

The themes from phase 2 qualitative data provided us with an outline of what subject areas (targets) the website should consider. The themes included:

- Feeling prepared and equipped
- feeling connected and supported
- valuing themselves as a caregiver and an individual
- maintaining control of the caring situation and being the co-ordinator of care

The caregiver theory from Parkinson and colleagues helped us refine these targets and frame the targets of the website in terms of building resilience [30]. The systematic review from phase 1 helped provide an evidence basis to identify some of the core components (i.e. peer support) to use on the website to address the targets identified by caregivers. All three sources of data (interviews, systematic review and theory) were synthesised by mapping against one another using tabulation (see Table 1). This provided an initial basis for the intervention.

#### Stage 2 – identifying intervention targets and components

Following analysis of phase 1 and 2 data (reported elsewhere [23, 24]) and tabulation, the research development group met to discuss the development of a prototype, using a modified nominal group process [28, 32–34]; a first step to co-production. Nominal group processes are structured meetings which have a specific problem to solve, facilitate group thinking and decision making. The group was tasked with aiding interpretation, synthesising the data and deciding upon the content of the prototype website. The key ideas and data from phases 1 and 2 were presented to the research development group using PowerPoint and handouts, highlighting key messages and ideas for an initial prototype. The group were asked specific questions to generate discussion, including the importance of the proposed core components and methods to address this, reflecting on the evidence presented and their experience and knowledge of the field. Members of the group were specifically asked to consider other interventions or support websites that we had not considered in the evidence synthesis including national and local organisations which provide similar support. Discussions were encouraged on how this study could learn from other organisations and how we could work collaboratively with such organisations to reduce duplication of work and efforts. Discussions were facilitated by two members of the research team (ND, SI) and detailed notes about discussions and agreements were taken by a third member of the team (NW). Variation in opinions and views was deliberately sought within the discussions by the facilitated and any disagreements were discussed until a consensus was reached about the content of the intervention.

#### Stage 3 – developing the intervention prototype

The outcome of the group meeting was a list of key targets and components for the intervention, matched with how these could be addressed by the intervention. This list was used to refine the initial table developed in stage 1, producing a list of targets and component as can be seen in Table 1. This tabulation of data informed the first iteration of the prototype.

The prototype of the website was developed using Microsoft PowerPoint by NW. The prototype website development focussed on producing an outline of what the content of the prototype website would include. Developing detailed textual information and the design of the interface was not a priority at this stage. After an initial draft was developed members of the team (ND, JH, NW, SI, KW, GR) met regularly to discuss and critique further the prototype website, making suggestions and recommendations for changes based on the notes from the group discussion and evidence from phases 1 and 2, taking an iterative approach to development.

Following the development of the initial prototype and internal discussions among the research team, follow up meetings were conducted with individual members of the research development group to ensure provider and user perspectives were incorporated. These meetings allowed further discussion of points raised in the initial group meeting and during the prototype website development among the research team to further refine the prototype website. Due to the rapid nature of the iterative development of the intervention, individual meetings ensured that we were able to keep pace with the speed of the project and its timelines, and were not delayed by attempts to meet all group members at once. These additional individual meetings allowed for a rapid and thorough approach to the intervention development and continued our co-production approach. Following each meeting a rapid iteration of the prototype was produced.

#### Stage 4 - user testing

Once the first version of the prototype was agreed upon by the research team it was tested with end users (11 family caregivers) individually. The prototype was displayed using Microsoft PowerPoint on a tablet and caregivers were asked to browse through the different pages and sections of the prototype. A ‘think aloud’ approach was used, whereby participants vocalised what they were thinking whilst performing tasks or solving problems (in this case viewing the prototype website) [35]. A topic guide was developed following advice from experts in e-health and user experience, focussing on the content and topics included within the prototype. For this study, user testing was seen as continuum of co-production and informed further iterations of the prototype website. This co-production approach and user testing continued until a final prototype was developed which all parties approved of.

### Ethics

Ethical permission was approved by London - Hampstead Research Ethics Committee (16/LO/1017) and received approval from the Health Research Authority (HRA). University College London ethics committee provided approval for participants recruited outside of the NHS (e.g. third sector) (3344/005). All participants provided informed written consent to participation.

## Results

**Fig. 2:**
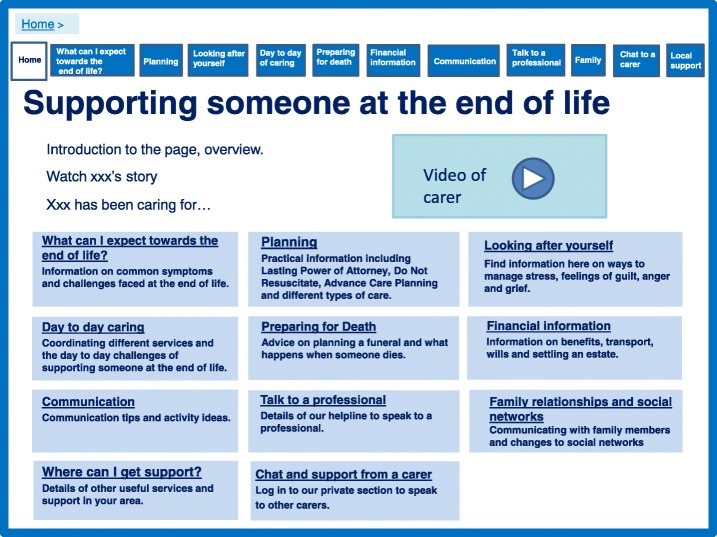
Website homepage

The prototype consisted of four main targets which caregivers found challenging: 1) feeling prepared and equipped; 2) feeling connected and supported; 3) valuing themselves as a caregiver and an individual; 4) maintaining control of the caring situation and being the co-ordinator of care. Within each of these targets we discuss the core intervention components to address these targets and relate these to the evidence from the systematic review, qualitative data and caregiver theory expanding the overview provided in Table 1. We provide images of the various pages of the prototype to illustrate how the four main targets are addressed. The homepage in Fig. 2 provides an overview of what is included.

### Participant characteristics

Participants recruited for user testing had a mean age of 74 years old and the majority were female (73%). A balance of spouses (55%) and adult children/children in law were recruited, and 55% were currently caring for someone. Participants were primarily white English. The majority of participants described their internet use as *daily* (*n* = 8), with 3 who described their usage as *weekly*. The majority of participants left education after the age of 20 years (*n* = 8), with two leaving between 17 and 20 years and one leaving before 15.

### Feeling prepared and equipped

**Fig. 3:**
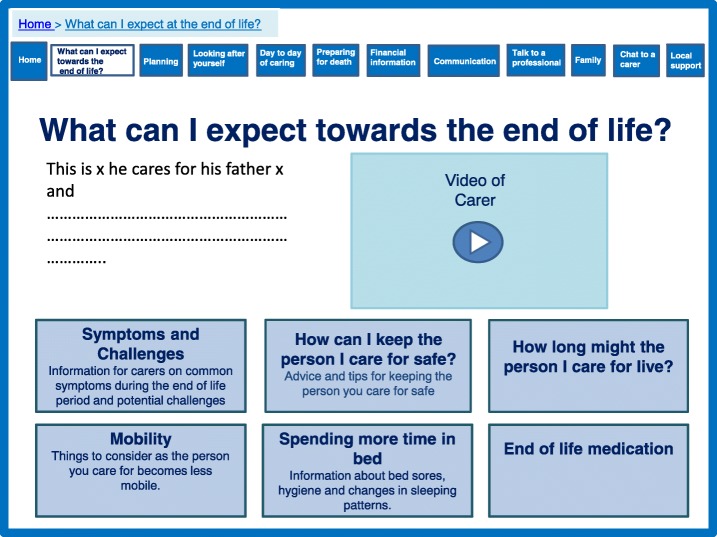
What can I expect towards the end of life?

**Fig. 4:**
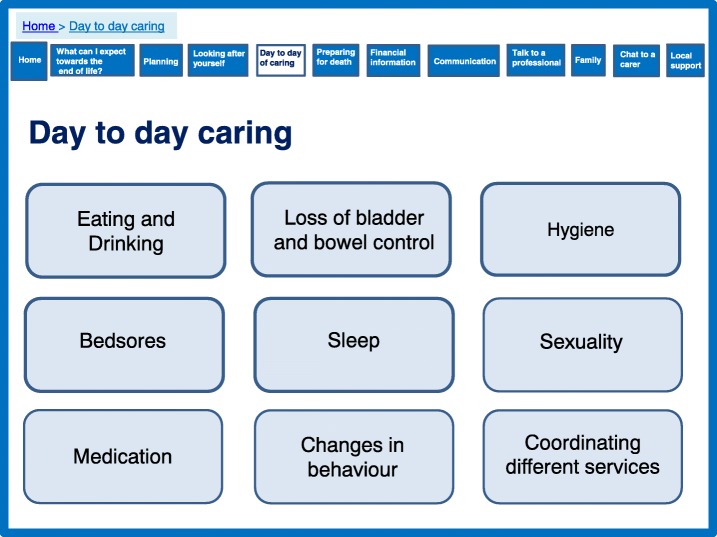
Day to day caring

**Fig. 5:**
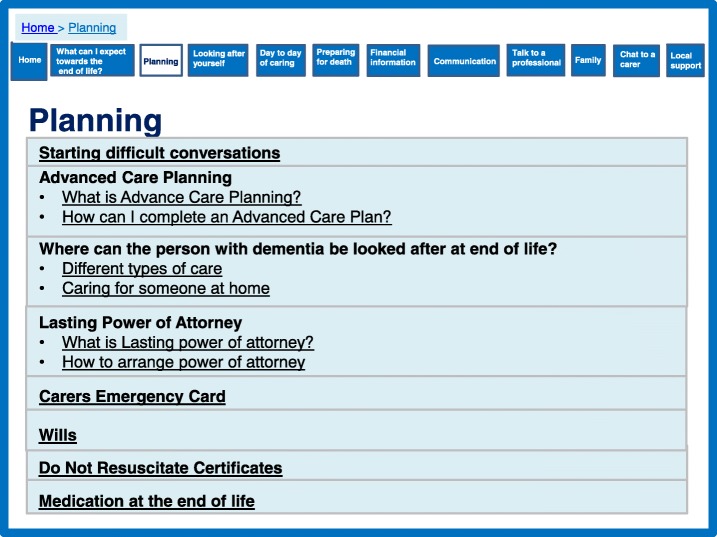
Planning for the future

The prototype website was designed to address ‘preparation’ and ‘feeling equipped’ throughout, but we provided some specific sections focussing on preparation (Figs. 3, 4, 5). The theory proposed that ensuring timely availability of key external resources is needed to support caregivers. External resources include relevant information and advice which can optimise support and prevent what the theory labels as ‘haphazard trial and error searches’ for assistance, which can create frustration. Figure 3 shows how these aspects of the prototype website were designed to create a sense of peer support by engaging the user through the use of videos of caregivers who families could relate to. Peer support was highlighted as important and effective in the systematic review and favoured in the interview data [16]. Further information was provided in this section to highlight expectations of physical and psychological decline with detail about symptoms, challenges, medication, behavioural changes, mobility and prognosis. Figure 4 demonstrates an additional section on day-to-day caring including being honest and realistic about what can be expected, including taboo areas such as incontinence and sexuality discussed by caregivers. These sections are complemented with a care planning section (Fig. 5), which focussed on the need for proactive thinking and planning as opposed to reactive crisis management.

### Feeling connected and supported

**Fig. 6:**
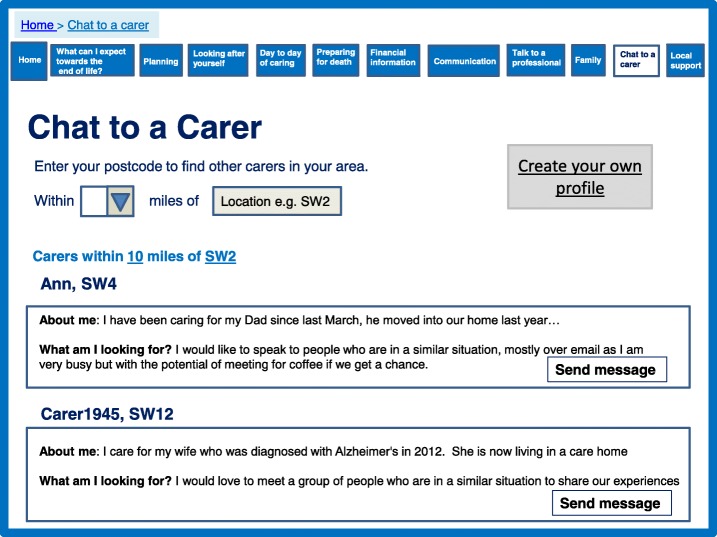
Chat to a caregiver

Participants in our qualitative study described caring as a lonely experience in which they often felt socially isolated. There was a need to feel both connected with other caregivers, but also supported by professionals. Our systematic review demonstrated that support could be provided online though peer interaction, in addition to contact with professionals. In particular opportunities to engage with other caregivers in the same situation appeared beneficial. Interaction in a group situation and the opportunity for visual contact appeared more promising than a simple ‘chat’ function and some studies indicated that peer support was associated with a reduction in stress, physical and emotional strain. The prototype directly addressed social networks and feeling connected by providing an opportunity to identify other caregivers for support and discussion, either privately or within a group (Fig. 6). We have importantly incorporated an aspect which allows caregivers to find other local caregivers. This addresses some caregivers’ need to meet in person and not simply chat online. We also incorporated a function for caregivers to create a profile which made the section much more personal and addressed some concerns participants had regarding privacy and not knowing with whom they may be talking.

Feeling connected and supported maps closely onto the theoretical theme of extending social assets for caregivers. The theory suggests that strong relational support networks can be effective support for caregivers, reinforcing their abilities to cope and acting as a protective factor against depression and moderating perceived quality of life. The theory also suggests that close links with other caregivers can reduce social isolation and depression.

**Fig. 7:**
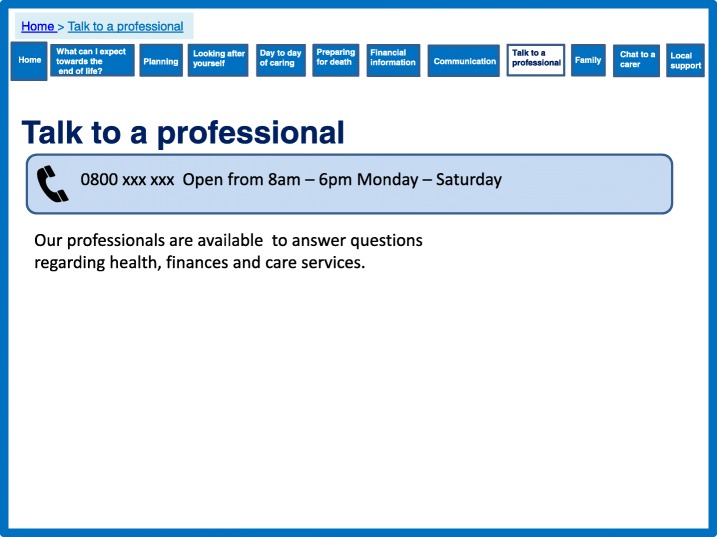
Talk to a professional

However, it was important for caregivers to feel confident in providing care and this confidence was sometimes sought from professionals. The theory states fostering effective service provider support is important, and that telephone calls can provide effective emotional support. There are several existing services which can provide this support via telephone and therefore the opportunity to integrate an existing telephone service was added into the development of the prototype website (Fig. 7).

**Fig. 8:**
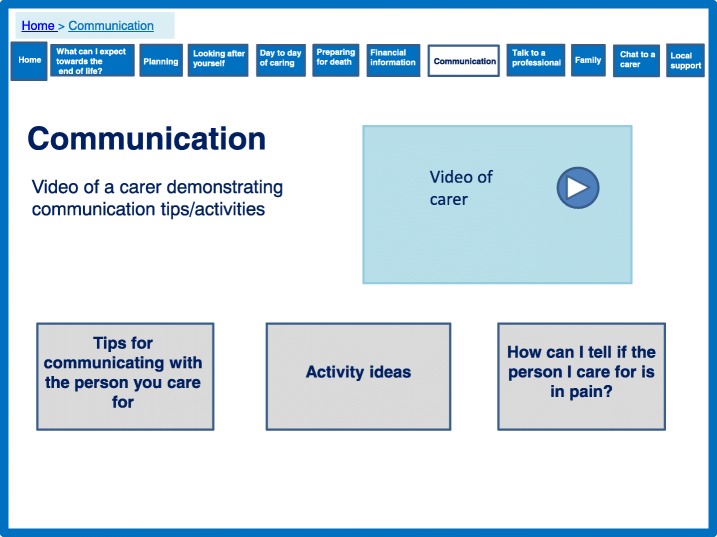
Communicating with the person with dementia

Finally, the importance of communication with the person with dementia which was highlighted in the qualitative data, was added to the development process. The prototype includes tips and advice on communicating with the person with dementia (Fig. 8). The theory states for extending social assets a good relationship with the person with dementia is important, and this has the potential to reduce behavioural problems which can increase caregiver stress.

### Valuing themselves as a caregiver and an individual

Participants in the qualitative study described an internal conflict about caring for themselves and maintaining their own life, whilst caring for and protecting the person with dementia, in some cases putting the person with dementia before their own health. The theory emphases the importance of valuing the caregiver, in particular focussing on strengthening key psychological resources available to them, maintaining the caregiver’s physical health status and safeguarding caregiver’s quality of life. To address these important factors and recognise the health and wellbeing of the caregiver we have blended functions of information, peer support and aspects of psychological support.

**Fig. 9:**
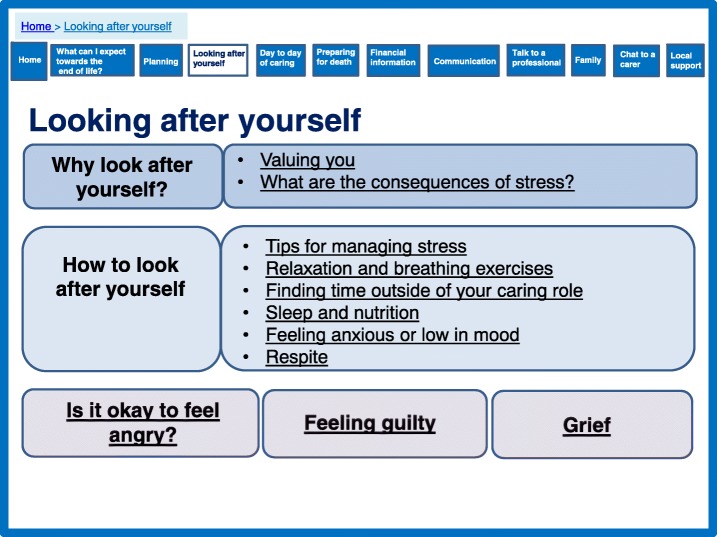
Looking after yourself

The prototype provides information about management and coping strategies. It also reassures caregivers about their feelings using experiences from other caregivers (Fig. 9) and functions of peer support (Fig. 6) to create a sense of shared experience. This addresses the importance of strengthening key psychological resources available to caregivers as identified by the theory. Figure 9 highlights how the prototype also address concerns of maintaining the caregiver’s physical health as identified in the theory.

**Fig. 10:**
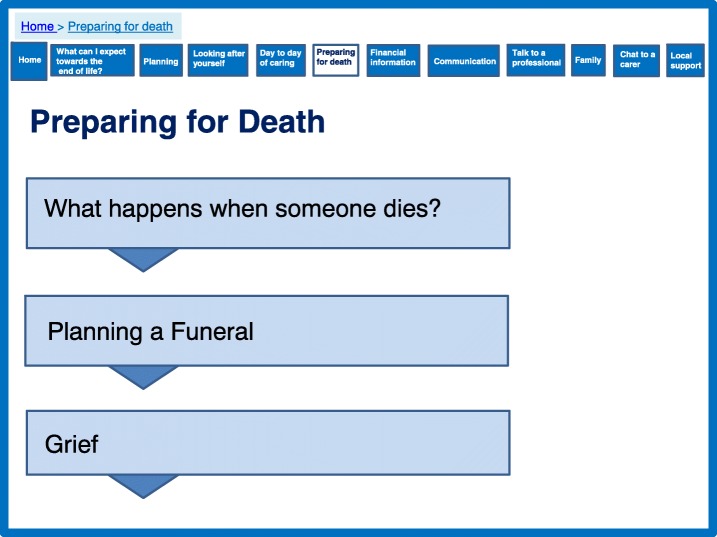
Preparing for death

In addition to looking after themselves and valuing themselves whilst caring, the prototype contains a section on preparing for the death of the person with dementia. Such post bereavement support, which was absent in the interventions identified in the systematic review, was identified as an important target in the qualitative data (Fig. 10).

### Maintaining control of the caring situation and being the coordinator of care

Caregivers felt that in order to receive the care their relative needed, they had to take control and manage this care. They described battles to negotiate and co-ordinate care, but also to receive financial assistance and aid through benefits and entitlements. Interspersed within discussions of control of care and the best interest of the individual with dementia, caregivers often discussed a lack of support from other family members with disagreements and difficult relationships within the wider family.

**Fig. 11:**
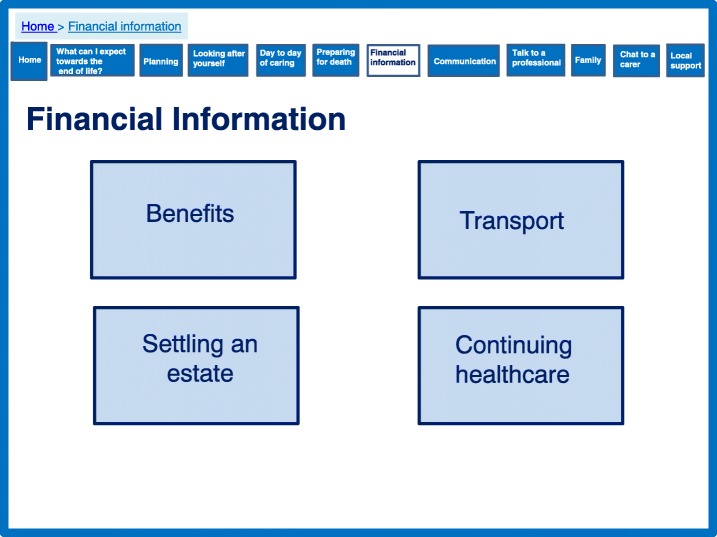
Financial information

**Fig. 12:**
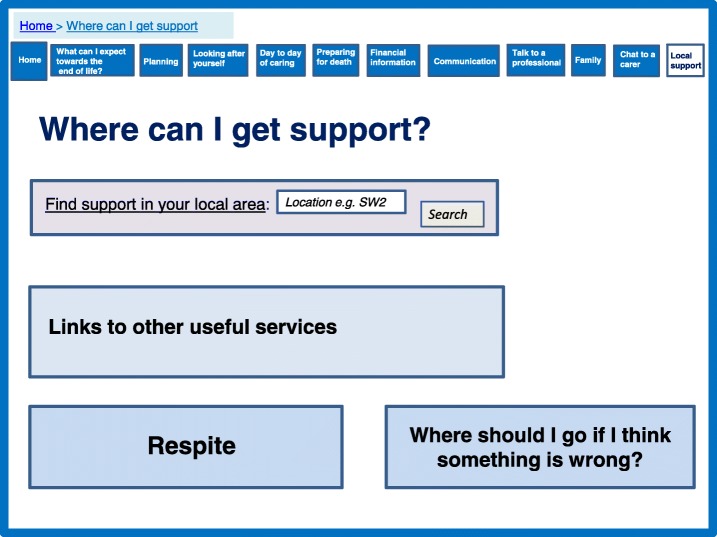
Local support

**Fig. 13:**
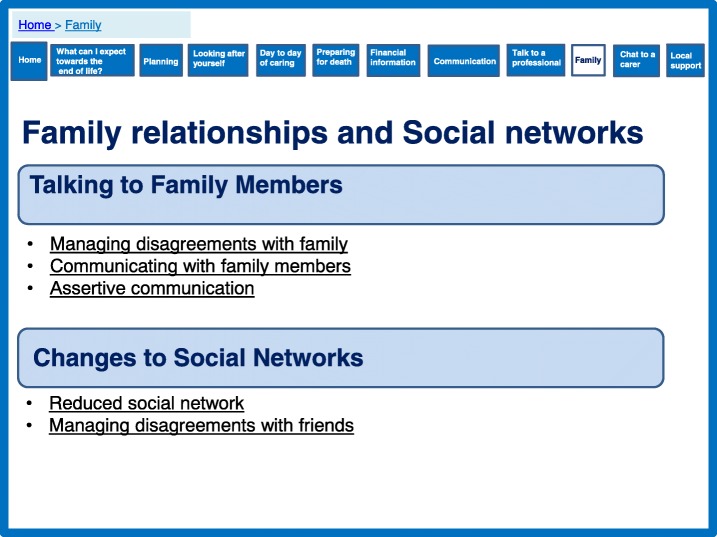
Family relationships and social networks

The prototype includes several features which are aimed to empower family caregivers. This includes providing information on financial support (Fig. 11) and signposting for local support services (Fig. 12), which were identified by the theory as important for caregiver support. We developed a section to encourage conversations among families to manage expectations and promote shared responsibility (Fig. 13). A lack of shared responsibility was highlighted within the qualitative data and the theory suggests strong relational support can reduce the number of hours single family members are engaged in daily care. This may have an impact on perceived quality of life and resilience.

## Discussion

A prototype website aimed to support family caregivers of someone with dementia towards the end of life in the UK was developed, informed by a systematic review, qualitative data, and a realist theoretical framework of what works to support family caregivers [30]. Reports of the early development of internet-based prototype interventions are limited in the health care literature generally, not just in dementia-related care. This paper provides a clear and transparent process for developing an internet-based intervention, providing an example for others developing digital or internet-based intervention.

The negative effects of caring for someone with dementia have been well reported, as well as the challenges which caregivers face including elevated levels of depressive symptoms, poorer physical health and decreased quality of life [36–38]. Several systematic reviews including the one published as part of the current study have demonstrated the benefits of internet-based interventions for caregivers living with adults with chronic conditions, including dementia [39]. However, there are few intervention studies which have focussed on supporting caregivers towards the end of life. This prototype not only helps to fill this gap of internet-based interventions, but also addresses concerns raised by caregivers about the lack of support reported in previous studies, including the qualitative data of this study [23, 24, 40–42].

As part of this prototype development we have incorporated the views of end users who suggested online support alone is not enough, there needs to be either face to face contact or telephone contact. Previous research has also reported the benefit of telephone support and telephone counselling, reducing depressive symptoms in caregivers of people with dementia [43]. The inclusion of professional support by adding a telephone support line to the prototype acknowledges that caregivers did not always want to hear other peoples’ problems when they had their own, and input from a professional was (for some) preferable [13].

Interviews with participants in the UK was used to inform the development of the prototype [23, 24], therefore reflecting the needs UK caregivers and services available to support them. However, many challenges within service and the needs of caregivers at the end of life are shared worldwide [13, 44], and the prototype could be applicable internationally, with some cultural translation. Data from a systematic review of international studies also supported the development of content and format [16].

### Strengths and weaknesses

A core strength of this study’s development process was the co-production methodology adopted with its bottom up approach to developing the prototype website. We used a definition of co-production in this study as an iterative process of developing a product (prototype website) with end users including caregivers and health care practitioners working closely with members of the research team [26, 27]. Representatives from both of these groups formed part of our research development group who oversaw and steered the development of the prototype. The content, structure and design of the prototype are grounded in the views and experiences of the end users. Adopting an iterative approach has ensured the content is acceptable and appropriate to this group. The development process was informed by recommendations from previous successful eHealth intervention guidance by placing more emphasis and time on the development of the initial ideas and content [45]. However, in contrast to previous co-production methods in similar studies we opted for a focus on individual meetings with end users (in this instance caregivers), as opposed to a series of panels or workshops. We chose this approach for several reasons. Firstly, end of life care and dementia are both sensitive topics, and testing and commenting on technology development can be seen as complex by some. Individual meetings minimised both the potential distress about sensitive topics and concerns about the complexity of the task in which they were engaged. Secondly, many of the caregivers who participated in this part of the research were still caring so their time was often limited and they were not able to leave home to travel to group meetings, therefore convening groups of caregivers was often difficult.

The co-productive and iterative nature of the website’s development is further strengthened by the use of evidence from a systematic review [16] and theoretical underpinnings [30]. The systematic review provided evidence of what components have previously been used in similar internet-based interventions, in particular those showing potential for effectiveness whilst being acceptable and useful. This informed the development process of how to address the targets of the intervention (i.e. peer support and telephone communication with professionals). The theoretical model from Parkinson and colleagues helped to focus the scope of the intervention, its targets and components identified in the qualitative data [30]. Although the theory was new when used to underpin the synthesis and focus of the intervention, it was derived from a realist review of the literature. Parkinson and colleagues explored what works to support family caregivers of people with dementia and followed an established realist methodology. This theory has been used to guide the development of a similar digital resource to support caregivers of older people [46].

It is important to recognise that a website will not reach all, and there are concerns that it can increase access to support whilst marginalising those who do not have access to the internet, increasing health inequalities [45]. However, other work has suggested that there was no inequality in access for other online interventions used in real practice [47]. If some caregivers use such a resource it might help to reduce the burden on health and social care resources, and free up resources which can be spent on those who are unable to use the internet.

Despite efforts to recruit family caregivers who did not use the internet at all or less frequently, few were engaged. Increasing the numbers in these categories would have helped us understand better how we can develop an intervention which would be more suitable for all. However, the sample was diverse in age and included a broad range of educational levels which can reflect internet usage and access. In addition to age being a factor in internet usage, previous research in older adults has shown that those with higher educational levels are more likely to use the internet [48, 49]. Therefore, it is possible that as ‘internet savvy’ generations age, age will become less of a factor influencing internet usage and education and language will remain a major factor influencing internet usage compared with age.

Finally, this study may have been strengthened by using more sophisticated software to develop the prototype rather than using Microsoft PowerPoint. More sophisticated software may have allowed for users to explore additional features and may have improved appearance.

### Lessons learnt for development

- Allowing time for iterations is vital in the development of all complex interventions; however the development of online interventions such as websites requires much more time and emphasis needs to be placed on the earlier phases of development.
- We suggest that due to time pressures and the iterative nature required for the development of online interventions a pragmatic approach to co-production may be needed for populations such as caregivers. Individual meetings with caregivers appears to be a valuable method for co-production and researchers should not assume that co-production panels and group meetings are required for robust methods. Individual meetings reduce the burden on caregivers and allows for fast paced intervention development, minimising delays to the overall research project.
- Synthesis of a variety of data sources is often not well reported in intervention development. The use of tabulation, adopting a matrix approach to display findings allows clear comparison of evidence.

### Implications for future research, policy and clinical practice

This is to our knowledge the first website, albeit prototype, based intervention which has been developed to support family caregivers of people with dementia at the end of life, with many other interventions focussing on the earlier and transition stages [50]. Further development of the prototype for a future trial will require emphasis on functionality, tailoring abilities and implementation, including organisational determinants of use, how carers would access and learn about the website and the wider context in which the prototype may later work. This has been neglected in previous online-based interventions aimed at family caregivers of people with dementia [51]. This intervention addresses many of the concerns of limited services available for family caregivers of people with dementia in end of life care phases, and concerns that end of life care policy simply focuses on the last days of life [13]. This prototype, developed into a full website has the potential to be useful to prepare family caregivers before the person they care for reaches the end of life stage, as well as after death and during bereavement. Many practitioners and caregivers of people with dementia still do not want to engage in difficult and significant conversations early enough to enable planning [40]. However, the need for information increases as the person with dementia approaches death and continues into bereavement [52]. A website such as this prototype could not only provide caregivers with information towards the end of life, but could also be a useful tool which helps caregivers to initiate discussions about future care and planning [53].

## Conclusions

This paper provides a detailed worked example of the development process of an internet-based intervention. The transparent methodology and key lessons learnt from developing the prototype should help those who are developing similar internet-based health interventions across conditions, not just dementia. Further, development of the prototype and trial is needed to explore the effect of the website and how it can be implemented in practice if effective.

## Data Availability

The datasets generated and/or analysed during the current study are not publicly available due to the data being qualitative data and ethical permissions restricting this but are available from the corresponding author on reasonable request.

## Abbreviations

FIG: Figure
JDR: Join Dementia Research
NHS: National Health Service
NIHR: National Institute for Health Research

## References

[1] Alzheimer’s Society. Dementia UK. 2nd ed. London: Alzheimer's Society; 2014.

[2] Alzheimer’s Society (2013). Carer Support.

[3] Department of Health (2012). Caring for our future.

[4] Kneebone II, Martin PR (2003). Coping and caregivers of people with dementia. Br J Health Psychol.

[5] Cuijpers P (2005). Depressive disorders in caregivers of dementia patients: a systematic review. Aging Ment Health.

[6] Ory MG, Hoffman RR, Yee JL, Tennstedt S, Schulz R (1999). Prevalence and impact of caregiving: a detailed comparison between dementia and nondementia caregivers. The Gerontologist.

[7] Hennings Jean, Froggatt Katherine (2016). The experiences of family caregivers of people with advanced dementia living in nursing homes, with a specific focus on spouses: A narrative literature review. Dementia.

[8] Brodaty H, Donkin M (2009). Family caregivers of people with dementia. Dialogues Clin Neurosci.

[9] Davies N, Rait G, Maio L, Iliffe S (2017). Family caregivers’ conceptualisation of quality end-of-life care for people with dementia: a qualitative study. Palliat Med.

[10] Davies N, Iliffe S. End of life care—why those with dementia have different needs. BMJ. 2016;353(i2171). 10.1136/bmj.i2171 27090749

[11] Alzheimer’s Society. Dementia 2015: aiming higher to transform lives. London: Alzheimer's Society; 2015.

[12] Poole M, Bamford C, McLellan E, Lee RP, Exley C, Hughes JC (2018). End-of-life care: a qualitative study comparing the views of people with dementia and family carers. Palliat Med.

[13] Broady TR, Saich F, Hinton T (2018). Caring for a family member or friend with dementia at the end of life: a scoping review and implications for palliative care practice. Palliat Med.

[14] Alzheimer’s Association. Alzheimer’s disease facts and figure. Chicago: Alzheimer's Association; 2018.

[15] Alzheimer’s Association (2014). 2014 Alzheimer’s disease facts and figures. Alzheimers Dement.

[16] Hopwood Jenny, Walker Nina, McDonagh Lorraine, Rait Greta, Walters Kate, Iliffe Stephen, Ross Jamie, Davies Nathan (2018). Internet-Based Interventions Aimed at Supporting Family Caregivers of People With Dementia: Systematic Review. Journal of Medical Internet Research.

[17] Whitlatch CJ, Orsulic-Jeras S (2018). Meeting the informational, educational, and psychosocial support needs of persons living with dementia and their family caregivers. Gerontologist.

[18] Boots L, Vugt M, Knippenberg R, Kempen G, Verhey F (2014). A systematic review of internet-based supportive interventions for caregivers of patients with dementia. Int J Geriatr Psychiatry.

[19] Chi N-C, Demiris G (2015). A systematic review of telehealth tools and interventions to support family caregivers. J Telemed Telecare.

[20] Hu C, Kung S, Rummans TA, Clark MM, Lapid MI (2014). Reducing caregiver stress with internet-based interventions: a systematic review of open-label and randomized controlled trials. J Am Med Inform Assoc.

[21] Kaltenbaugh Donna, Klem Mary Lou, Hu Lu, Turi Eleanor, Haines Alice, Hagerty Lingler Jennifer (2015). Using Web-Based Interventions to Support Caregivers of Patients With Cancer: A Systematic Review. Oncology Nursing Forum.

[22] Office for national Statistics. Statistical bulletin: Internet users in the UK: 2017. London: Office for National Statistics; 2017.

[23] Davies Nathan, Walker Nina, Hopwood Jenny, Iliffe Steve, Rait Greta, Walters Kate (2019). A “separation of worlds”: The support and social networks of family carers of people with dementia at the end of life, and the possible role of the internet. Health & Social Care in the Community.

[24] Davies N, Iliffe S, Hopwood J, Walker N, Ross J, Rait G, et al. The key aspects of online support that older family carers of people with dementia want at the end of life: a qualitative study. Aging Ment Health. 2019;29:1–8 10.1080/13607863.2019.1642299 31353937

[25] Radbruch L, Payne S (2009). White paper on standards and norms for hospice and palliative care in Europe: part 1. Eur J Palliat Care.

[26] Greenhalgh T, Jackson C, Shaw S, Janamian T (2016). Achieving research impact through co-creation in community-based health services: literature review and case study. Milbank Q.

[27] Ward M, De Brún A, Beirne D, Conway C, Cunningham U, English A (2018). Using co-design to develop a collective leadership intervention for healthcare teams to improve safety culture. Int J Environ Res Public Health.

[28] Davies N, Mathew R, Wilcock J, Manthorpe J, Sampson EL, Lamahewa K, et al. A co-design process developing heuristics for practitioners providing end of life care for people with dementia. BMC Palliat Care. 2016;15(68):1182. 10.1186/s12904-016-0146-z PMC4969644 27484683

[29] Craig P, Dieppe P, Macintyre S, Michie S, Nazareth I, Petticrew M (2008). Developing and evaluating complex interventions: the new Medical Research Council guidance. BMJ.

[30] Parkinson M, Carr S, Rushmer R, Abley C (2016). Investigating what works to support family carers of people with dementia: a rapid realist review. J Public Health.

[31] Brown MA, Sampson EL, Jones L, Barron AM (2013). Prognostic indicators of 6-month mortality in elderly people with advanced dementia: a systematic review. Palliat Med.

[32] Van de Ven AH, Delbecq AL (1972). The nominal group as a research instrument for exploratory health studies. Am J Public Health.

[33] Bartunek JM, Murningham JK (1984). The nominal group technique: expanding the basic procedure and underlying assumptions. Group Organ Stud.

[34] Davies N, Manthorpe J, Sampson E L, Iliffe S (2015). After the Liverpool Care Pathway—development of heuristics to guide end of life care for people with dementia: protocol of the ALCP study: Figure 1. BMJ Open.

[35] Ericsson KA, Simon HA (1993). Protocol analysis. Verbal reports as data.

[36] Etters L, Goodall D, Harrison BE (2008). Caregiver burden among dementia patient caregivers: a review of the literature. J Am Acad Nurse Pract.

[37] Perkins M, Howard VJ, Wadley VG, Crowe M, Safford MM, Haley WE (2012). Caregiving strain and all-cause mortality: evidence from the REGARDS study. J Gerontol B Psychol Sci Soc Sci.

[38] Pinquart M, Sörensen S (2006). Helping caregivers of persons with dementia: which interventions work and how large are their effects?. Int Psychogeriatr.

[39] Sherifali Diana, Ali Muhammad Usman, Ploeg Jenny, Markle-Reid Maureen, Valaitis Ruta, Bartholomew Amy, Fitzpatrick-Lewis Donna, McAiney Carrie (2018). Impact of Internet-Based Interventions on Caregiver Mental Health: Systematic Review and Meta-Analysis. Journal of Medical Internet Research.

[40] Davies N, Maio L, Rait G, Iliffe S (2014). Quality end-of-life care for dementia: what have family carers told us so far? a narrative synthesis. Palliat Med.

[41] Lewis L (2014). Caregivers’ experiences seeking hospice care for loved ones with dementia. Qual Health Res.

[42] Caron CD, Griffith J, Arcand M (2005). Decision making at the end of life in dementia: how family caregivers perceive their interactions with health care providers in long-term-care settings. J Appl Gerontol.

[43] Lins S, Hayder-Beichel D, Rücker G, Motschall E, Antes G, Meyer G, et al. Efficacy and experiences of telephone counselling for informal carers of people with dementia. Cochrane Libr. 2014;1(9):CD009126. 10.1002/14651858.CD009126.pub2 PMC7433299 25177838

[44] Davies N, Maio L, Van Riet PJ, Mariani E, Jaspers B, Sommerbakk R (2013). Quality palliative care for cancer and dementia in five European countries: some common challenges. Aging Ment Health.

[45] Murray Elizabeth (2012). Web-Based Interventions for Behavior Change and Self-Management: Potential, Pitfalls, and Progress. Medicine 2.0.

[46] Dale J, Loew J, Nanton V, Grason SG (2018). Coproduction of a theory-based digital resource for unpaid Carers (the care companion): mixed-methods study. JMIR Aging.

[47] Poduval S, Ahmed S, Marston L, Hamilton F, Murray E (2018). Crossing the digital divide in online self-management support: analysis of usage data from HeLP-diabetes. JMIR Diabetes.

[48] Arcury TA, Sandberg JC, Melius KP, Quandt SA, Leng X, Latulipe C (2018). Older adult internet use and eHealth literacy. J Appl Gerontol.

[49] Neter E, Brainin E (2012). eHealth literacy: extending the digital divide to the realm of health information. J Med Internet Res.

[50] Dickinson C, Dow J, Gibson G, Hayes L, Robalino S, Robinson L (2017). Psychosocial intervention for carers of people with dementia: what components are most effective and when? a systematic review of systematic reviews. Int Psychogeriatr.

[51] Christie Hannah L., Bartels Sara L., Boots Lizzy M.M., Tange Huibert J., Verhey Frans R.J., de Vugt Marjolein E. (2018). A systematic review on the implementation of eHealth interventions for informal caregivers of people with dementia. Internet Interventions.

[52] Muders P, Zahrt-Omar CA, Bussmann S, Haberstroh J, Weber M (2015). Support for families of patients dying with dementia: a qualitative analysis of bereaved family members’ experiences and suggestions. Palliat Support Care.

[53] Hughes JC, Volicer L, van der Steen JT (2018). Complexity and gaps: the high-hanging fruit of dementia and palliative care research. Palliat Med.

